# Vacuum/Compression Infiltration-mediated Permeation Pathway of a Peptide-pDNA Complex as a Non-Viral Carrier for Gene Delivery *in Planta*

**DOI:** 10.1038/s41598-018-36466-1

**Published:** 2019-01-22

**Authors:** Keiko Midorikawa, Yutaka Kodama, Keiji Numata

**Affiliations:** 1 Biomacromoleules Research Team, RIKEN Center for Sustainable Resource Science, 2-1 Hirosawa, Wako-shi, Saitama 351-0198 Japan; 2 Center for Bioscience Research and Education, Utsunomiya University, Tochigi, 321-8505 Japan

## Abstract

Non-viral gene carriers have been extensively investigated as alternatives to viral vectors for gene delivery systems into animal and plant cells. A non-viral gene carrier containing a cell-penetrating peptide and a cationic sequence was previously developed for use in intact plants and plant cells; however, the permeation pathway of the gene carrier into plant cells is yet to be elucidated, which would facilitate the improvement of the gene delivery efficiency. Here, we identified the vacuum/compression infiltration-mediated permeation pathway of a non-viral gene carrier into plant tissues and cells using a complex of plasmid DNA and a peptide-based gene carrier. This complex was taken up via the hydathodes in *Arabidopsis thaliana*, and from root hairs in *Nicotiana benthamiana*. Remarkably, these structurally weak tissues are also routes of bacterial invasion in nature, suggesting that peptide-pDNA complexes invade intact plants through similar pathways as bacterial pathogens.

## Introduction

Transgenic plant technologies are necessary for producing useful value-added substances from plant metabolites. In particular, the development of genome-editing systems such as CRISPR/Cas9 and TALEN has enabled the stricter control of gene expression^1,2^. In addition to these technological advances, delivery systems for transferring genetic modification modules into plant cells has been developed^3^. The presence of the cell wall surrounding plant cells prevents the invasion of foreign matter, leaving researchers with relatively few options for the delivery of functional biomolecules and genetic material into plant cells^4^. The *Agrobacterium* method commonly used to transform many dicot plant species is not applicable to several monocots because they are not infectible by this bacterium, limiting the number of plant species that can be transformed in this way.

Non-viral carriers, which can be used to transform any plant species without specialized equipment, are an effective alternative to overcome species-specific transformation problems. The use of non-viral carriers such as cationic liposomes or polycations was previously described in various studies^5–9^. Ionic complexes of polycations and negatively charged nucleic acids penetrate the plasma membrane via electrostatic interactions. Additionally, studies using cell-penetrating peptides (CPPs) as a non-viral carrier have also been reported^10^. In recent years, the ability of CPPs to deliver macromolecules such as DNA, RNA, and protein into living cells of various plants has also been investigated^11–14^, and more recently, the use of a carrier combining CPP and carbon nanotubes has also been reported^15^. Similarly, in our previous study, a fusion peptide consisting of a CPP and a cationic sequence interacting with a nucleic acid was used as a non-viral carrier to introduce nucleic acids into plant cells^16^. CPP can pass through the plasma membranes of both animal and plant cells, despite their different lipid compositions^17^. Bp100 (amino acid sequence: KKLFKKILKYL) is an amphipathic CPP that can pass through the membrane in the structure of an α-helix, and was originally optimized as an antimicrobial peptide against plant pathogens^18^. In addition, we previously added a random coil sequence (KH_9_) consisting of lysine and histidine^19^ as a polycation to Bp100, which facilitated its interaction with plasmid DNA (pDNA)^16^. In the presence of this cationic sequence, the pDNA is condensed, resulting in the formation of a complex between the fusion peptide and the pDNA, held together by the ionic interaction between the molecules^20^. Histidine, one of the cationic amino acids, was also reported to facilitate release of complexes from the vesicles by proton sponge effect which destabilized endosomes^21^. Using fusion peptide-mediated transformation, the peptide-pDNA complex was incorporated into the leaf cells of *Arabidopsis thaliana* and *Nicotiana benthamiana*^16^, and dsRNA and proteins were also able to be delivered into cells using this method^22,23^. Furthermore, fusion peptides containing a mitochondrial- or chloroplast-targeting signal enable organelle-specific gene delivery^24,25^.

Fusion peptide-mediated transformation is a very versatile technology that overcomes the problems of the commonly used transformation approaches; nevertheless, further improvement in transformation efficiency is required before the technique can be routinely used in crop breeding and plant biotechnology. To improve the efficiency of cargo delivery, selecting the optimal tissue for infiltration by the peptide-pDNA complex is critical; however, the uptake and transport pathways of these complexes into plant cells have not yet been elucidated. Here, we investigated the vacuum/compression infiltration pathways of a peptide-pDNA complex into *A. thaliana* and *N. benthamiana*, showing that the complex is taken up by multiple tissues. Confocal laser scanning microscopy (CLSM) revealed that the uptake of the complexes in *A. thaliana* mainly occurred via the hydathodes in the leaves, whereas the uptake in *N. benthamiana* occurred via the root hairs. These plant tissues are structurally weak regions that are also targeted by bacterial invasion pathways. This research provides a new insight into the relationship between bacterial infection and non-viral gene delivery systems in plant cells, and could facilitate the enhancement of the transformation efficiency of various plant species.

## Results

### Quantitative permeation of the peptide-pDNA complex into the shoot and root tissues

**Figure 1:**
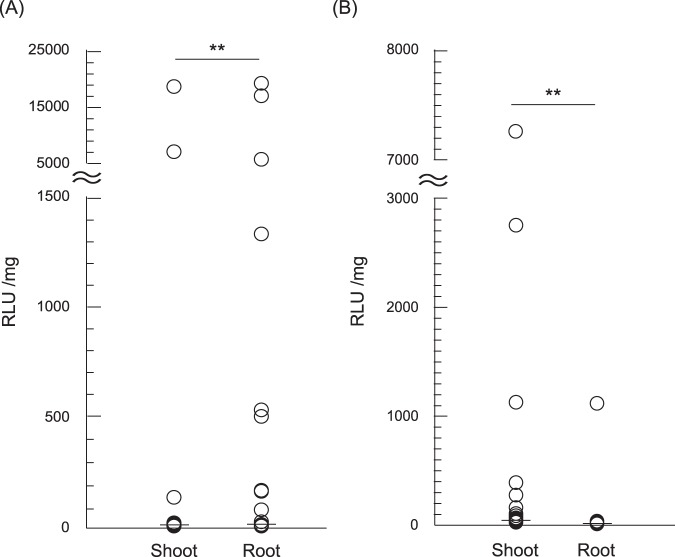
Evaluating the permeation of the peptide-based DNA carriers into different seedling tissues. The peptide-pDNA complex consisting of Bp100(KH)_9_ and 35S-Nluc-TNOS was transformed into seedlings by degassing and pressurizing them. The transformation efficiency of *N. benthamiana* (**A**) and *A. thaliana* (**B**) 7-DAG seedlings was compared in the shoots and roots using an Nluc assay (n = 83, **p < 0.01, Mann-Whitney *U* test).

For the evaluation of the vacuum/compression infiltration-mediated permeation of the peptide-pDNA complex into different tissues of plant seedlings, we prepared an ionic complex of pDNA encoding NanoLuc luciferase (Nluc) with Bp100(KH)_9_ as a CPP-containing fusion peptide. The diameter and zeta potential (surface electrical charge) of the complex were measured using dynamic light scattering (DLS) and a zeta-nanosizer, respectively (Supplementary Table 1). The peptide-pDNA complex was infiltrated into *N. benthamiana* and *A. thaliana* (accession: Col-0) seedlings at seven days after germination (DAG) by degassing and pressurizing seedlings immersed in the complex solution. We then measured the respective Nluc activities of the shoots and roots of the seedlings. The groups were statistically analyzed using a Mann-Whitney *U* test because of the non-parametric distribution of the data. We considered samples whose relative light units (RLU) value were beyond the interquartile range of each group by a factor of 1.5 or more to be significantly transfected. In *N. benthamiana*, three of the 83 samples had a high Nluc activity in the shoot, while eight had an increased Nluc activity in the roots, with a greater activity in the root cells than the shoots overall (Fig. 1A). In contrast, the Nluc activity in *A. thaliana* was higher in the shoots than the roots, with 10 of 83 samples demonstrating high Nluc activity in the shoot (Fig. 1B). Only one sample had high Nluc activity in the root, which could have been transformed following the infiltration of a wound on the root. These comparisons indicated that the peptide-pDNA complex entered intact *N. benthamiana* and *A. thaliana* plants via different permeation pathways.

### Permeation pathway in *N. benthamiana*

**Figure 2:**
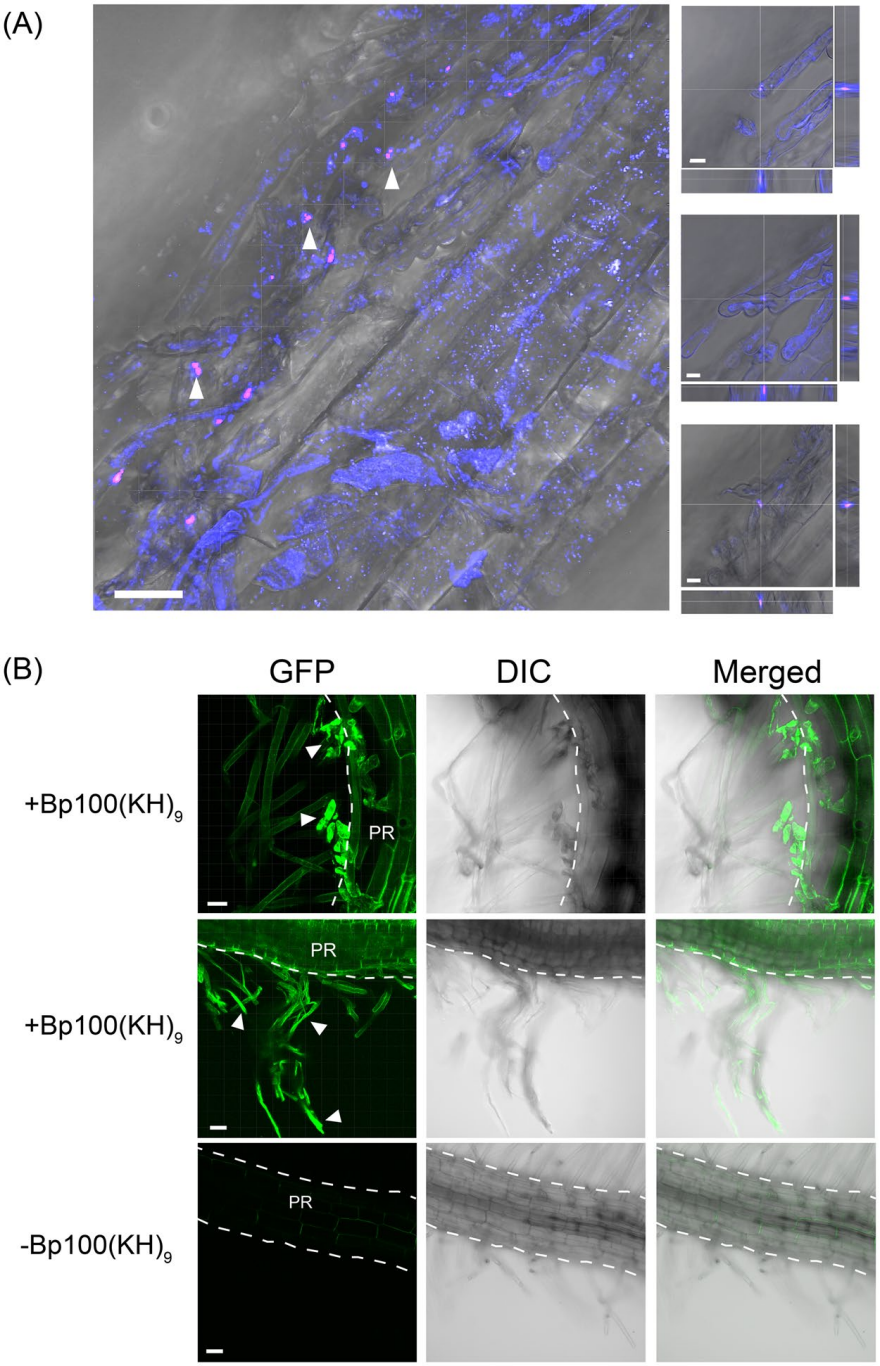
Uptake of the peptide-pDNA complex and expression in *N. benthamiana* roots. (**A**) A complex consisting of Cy3-labeled pDNA (35S-Nluc-TNOS) and the fusion peptide, Bp100(KH)_9_ was infiltrated into 7-DAG seedlings. pDNA labeled with Cy3 is shown in magenta and the plasma membrane or vesicles stained with FM4-64 are shown in blue. The complexes were observed around the root hairs. The arrowheads indicate the regions containing the complex of Cy3-labeled pDNA (magenta). The regions indicated by the arrowheads were enlarged and obtained at different focus on the right, with the Z stack longitudinal sections on the right of the enlarged views and the transverse sections at the bottom. The CLSM observations were performed within one hour of M. Scale bars represent 30 μm on the left, 8 μm on the right. (**B**) GFP fluorescence (green) in *N. benthamiana* roots following infiltration with the peptide-based gene carrier. The upper and middle rows are samples into which a peptide-pDNA complex consisting of Bp100(KH)_9_ and 35S-GFP(S65T)-TNOS was infiltrated. The bottom is a sample in which only pDNA was infiltrated as a control. The white arrowheads indicate the root hair part expressing GFP. The outline of the primary root (PR) is indicated by a dotted line. Scale bars represent 30 μm.

Since *N. benthamiana* had a high transformation efficiency in the root tissues, the intracellular distribution of the peptide-pDNA complex in the root was investigated using CLSM. The *N. benthamiana* seedlings were vacuum/compression infiltrated with complexes consisting of a Cy3-labeled pDNA and the fusion peptide Bp100(KH)_9_, using the method outlined above. To confirm that the complex was incorporated into the cytoplasm, both the plasma membrane and the vesicles in living cells were labeled with FM4-64. CLSM observations were performed within one hour of the degassing and pressurizing treatments. More Cy3 signal was observed in the vicinity of the root hairs than in the primary root (Fig. 2A). Additionally, a confocal Z-stack analysis confirmed the presence of the Cy3 signal inside the root hairs (Fig. 2A, enlarged images). The uptake of Cy3-labeled pDNA was confirmed in all the seedlings examined. A further CLSM imaging analysis was performed on *N. benthamiana* seedlings transformed with a complex containing a pDNA encoding GFP, revealing the presence of the GFP fluorescence signal in the root epidermal cells (Fig. 2B). These results showed that the peptide-pDNA complexes are incorporated into *N. benthamiana* seedlings via the root hairs.

Unlike the roots, *N. benthamiana* shoots were not highly permeable to the peptide-pDNA complex (Fig. 1A). We detected the Cy3 signal of the labeled pDNA in the stomatal pores (Supplementary Fig. 1A). However, similar to the Nluc assay results, no GFP fluorescence was observed in the leaves. Therefore, in order to further investigate the permeability of the complex solution by degassing and pressurizing treatment, we infiltrated the Rhodamine B solution into *N. benthamiana* leaves in the same method as the infiltration of the complex solution (Supplementary Fig. 1B). As a result, little penetration of the dye into the leaves following the degassing and pressurizing treatments was detected. These results confirmed that the peptide-pDNA complex does not easily permeate into the *N. benthamiana* leaf cells during the degassing and pressurizing treatments.

### Permeation pathway in *A. thaliana*

**Figure 3:**
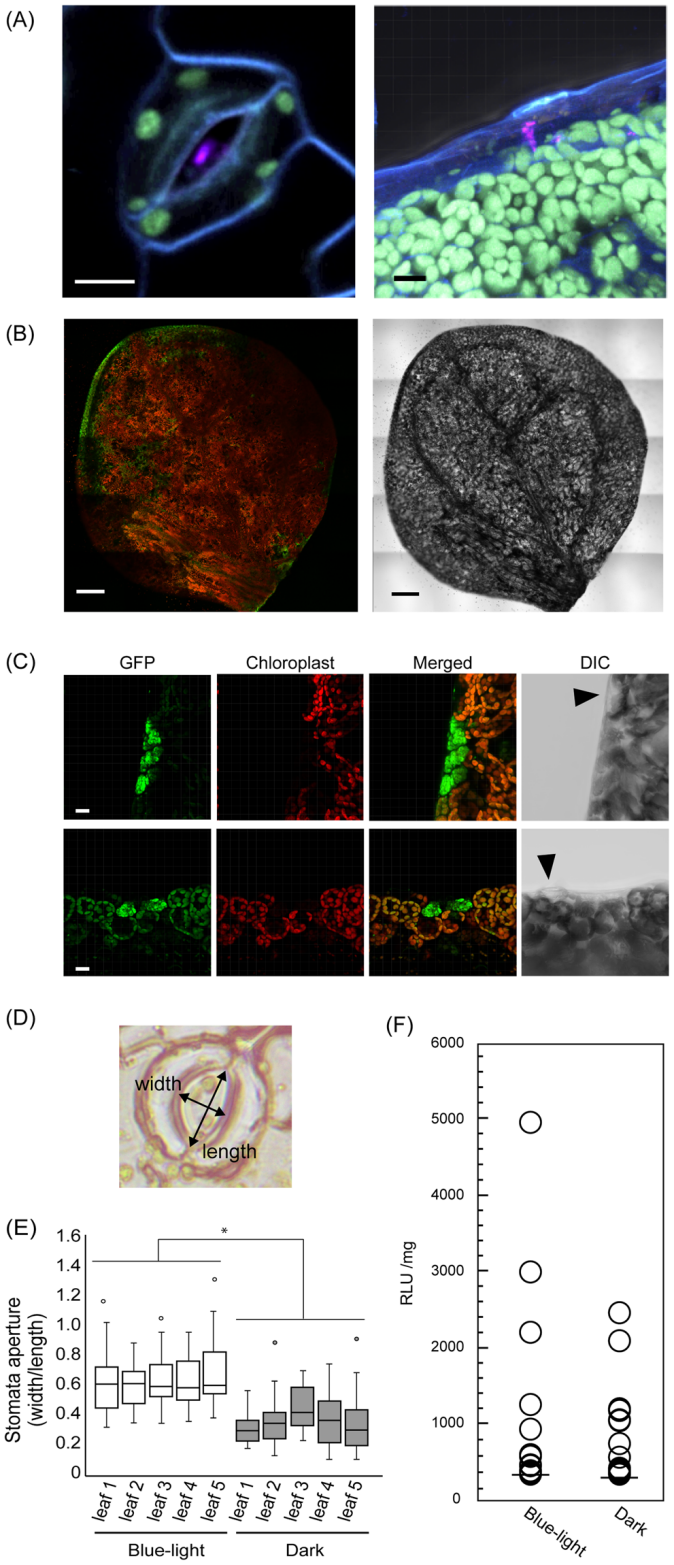
Uptake of the peptide-pDNA complex and *A. thaliana* leaves. (**A**) The complex was observed in stomata (left) or hydathodes (right). Complexes consisting of Cy3-labeled pDNA (35S-Nluc-TNOS) and the fusion peptide Bp100(KH)_9_ were infiltrated into 7-DAG seedlings. pDNA labeled with Cy3 is shown in magenta, chloroplast autofluorescence is green, and the plasma membrane stained with FM4-64 is shown in blue. Scale bars are 10 μm. (**B**) The whole leaf of *A. thaliana* was infiltrated with the peptide-pDNA complex consisting of Bp100(KH)_9_ and 35S-GFP(S65T)-TNOS. CSLM was used to observe the leaves 24 h after the degassing and pressurizing treatments. GFP fluorescence (green) was mainly detected at the leaf edge (left). Red is chloroplast autofluorescence. The right image shows DIC. Scale bars are 200 μm. (**C**) GFP (green) fluorescence in the leaf margin of *A. thaliana* differs from chloroplast autofluorescence (red). The upper and lower panels are representative examples. Arrowheads in DIC indicate hydathodes. Scale bars are 15 μm. (**D**) Relative stomatal aperture was calculated by dividing the width of the stomatal pore by its length. (**E**) Relationship between the relative stomatal apertures of *A. thaliana* seedlings caused by different light conditions (blue light or darkness) and the efficiency of peptide-pDNA complex penetration into the leaf. Box plots show the median and 25th and 75th percentiles; whiskers represent confidence intervals, and open circles indicate outliers (n = 30, *p < 0.05, *t*-test, mean ± SD). (**F**) An Nluc assay performed under the two light conditions using 14-DAG *A. thaliana* seedlings. Total proteins were extracted from the shoots only (n = 56, *p* = 0.821, Mann-Whitney *U* test).

We also investigated the vacuum/compression infiltration pathway of the complex into *A. thaliana*. CLSM observations revealed that the Cy3-labeled pDNA was localized in the stomata and water pores in the *A. thaliana* leaves (Fig. 3A). The water pores are part of the hydathode structures at the edges of leaves, which enable the drainage of excess xylem fluid in the process of guttation. Based on the observation of the Cy3 signal localization, we predicted that the uptake of the peptide-pDNA complexes occurred via the stomata and hydathodes; however, our CLSM observations revealed that most of the GFP fluorescence signal was localized at the edge of the leaf (Fig. 3B). The merged images of the chloroplast autofluorescence and the GFP fluorescence signals clearly showed that GFP was not localized to the epidermal cells; rather, the GFP fluorescence was detected in the cells around the hydathodes, known as the epithem cells (Fig. 3C). These epithem cells are part of the group of cells that form below the hydathode pores, suggesting that the peptide-pDNA complex was mainly incorporated into the *A. thaliana* leaves via the hydathodes.

Although Cy3-labeled pDNA was observed in the stomatal pores (Fig. 3A), very little GFP fluorescence was detected in the epidermal cells of the *A. thaliana* leaves. In the vicinity of the stomatal pore, complexes containing the Cy3-labeled pDNA were also observed in the intercellular space (Supplementary Fig. 2A,B). From these results, we hypothesized that the complex accumulated in the intercellular space without passing through the cell walls of the epidermal and mesophyll cells. To confirm this, we investigated the contribution of the stomatal pores to the permeability of the peptide-pDNA complex. Stomata are known to open under blue-light irradiation and close in darkness^26^. Exploiting this property, *A. thaliana* seedlings treated under the two different light conditions (blue light and darkness) were subjected to the Nluc assay following vacuum/compression infiltration with the peptide-pDNA complex. We predicted that the transformation efficiencies would differ between seedlings grown in these two light conditions because of the different incorporations of the complex via the stomata. The relative stomatal apertures were strikingly different between the *A. thaliana* seedlings incubated under blue light and darkness for three hours (Fig. 3D,E). Following the degassing and pressurizing treatments of seedlings in the complex solution, the Nluc activities of the shoots in the two light conditions were not significantly different (Fig. 3F), suggesting that their different relative stomatal apertures did not affect the transformation efficiency mediated by the fusion peptide carrier. When Rhodamine B solution was infiltrated into *A. thaliana* leaves using the degassing and pressurization treatments, the solution was clearly observed to penetrate the leaf via the hydathodes, in contrast with the *N. benthamiana* leaves (Supplementary Fig. 3). These results strongly suggested that the peptide-pDNA complex infiltrated *A. thaliana* leaves via the hydathodes.

### Uptake of the peptide-pDNA complex via the root hairs

**Figure 4:**
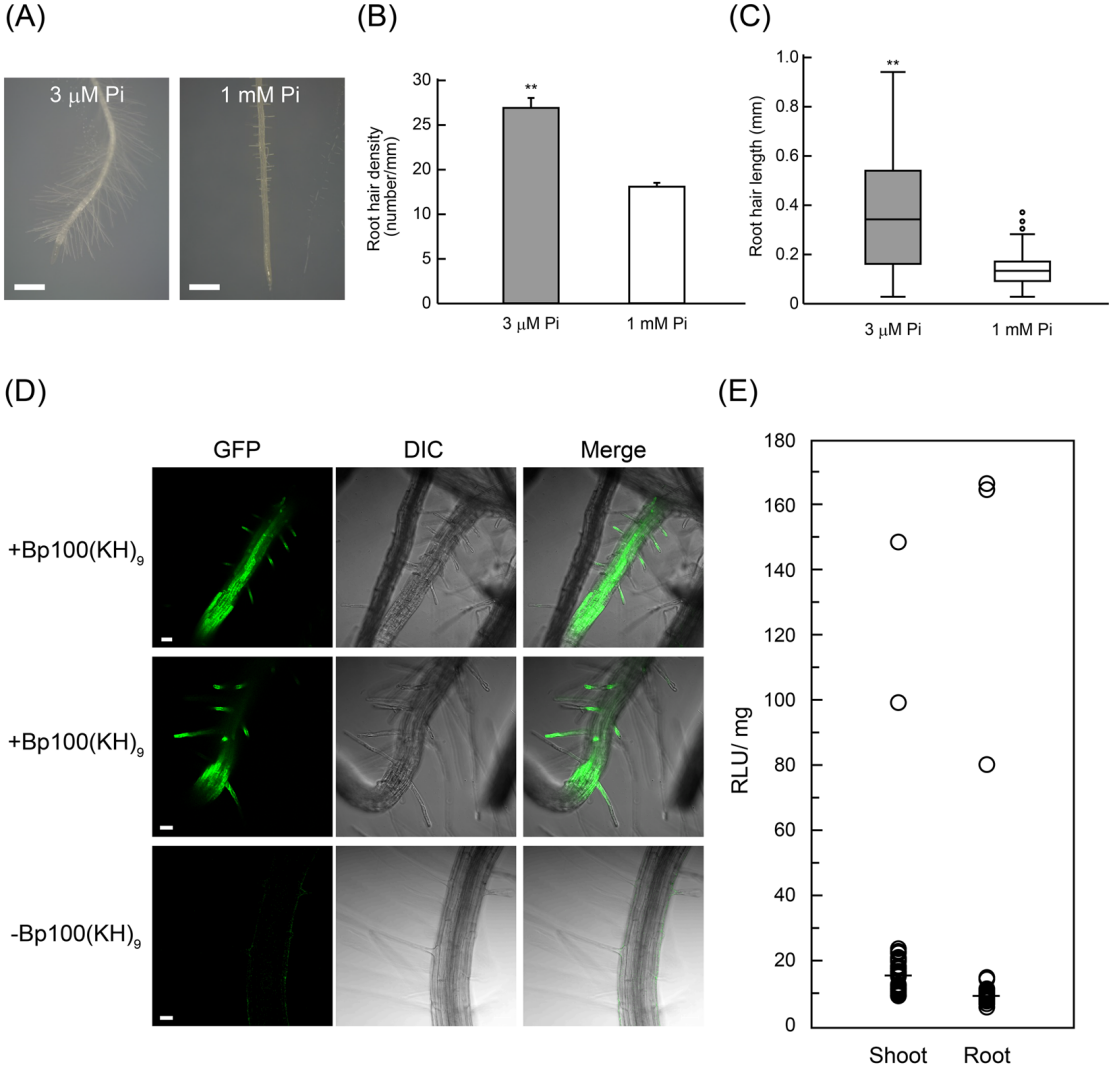
Evaluation of the contribution of root hairs to the uptake of the peptide-pDNA complex. (**A**) The roots of 7-DAG *A. thaliana* seedlings grown under phosphate-sufficient (1 mM Pi) and -deficient (3 μM Pi) conditions. Scale bars represent 100 μm. (**B**) Root hair densities and (**C**) lengths under the two phosphate conditions, measured in the region 0 to 5 mm from the root tip. Box plots show the median and 25th and 75th percentiles; whiskers represent confidence intervals, and open circles indicate outliers (n = 3, **p < 0.01, *t*-test, mean ± SD). (**D**) Microscopic evaluation of the transformation efficiencies of seedlings under the phosphate-deficient condition, using *GFP* as a reporter gene. The upper and middle rows are samples into which a peptide-pDNA complex consisting of Bp100(KH)_9_ and 35S-GFP(S65T)-TNOS was infiltrated. The bottom is a sample into which only pDNA was infiltrated as a control. Scale bars represent 50 μm. (**E**) An Nluc assay in *A. thaliana* grown under the phosphate-deficient condition (n = 40, *p* = 0.777, Mann-Whitney *U* test).

To elucidate the contribution of membrane transport to the uptake of the peptide-pDNA complex, the plasma membrane and vesicles were stained with FM4-64. In *A. thaliana*, FM4-64 staining showed that the membrane transport of the complex was weak in the root cells, and no Cy3 signal was observed around the root hairs (Supplementary Fig. 4). In comparison with *N. benthamiana*, *A. thaliana* has less developed root hairs and quite a different root architecture (Supplementary Fig. 5A,B). We investigated the effect of root hair development on the permeation and transformation efficiencies of the peptide-pDNA complex in root cells. The exposure of plants to phosphate (Pi)-deficient conditions was previously reported to promote root hair development^27^. We therefore grew *A. thaliana* seedlings on a phosphate-deficient medium (3 μM phosphate) to increase their root hair length and density, in addition to growing seedlings under normal conditions (1 mM phosphate) (Fig. 4A). The density and length of root hairs were markedly enhanced under the phosphate-deficient conditions (Fig. 4B,C), and these seedlings were used to evaluate the transformation efficiency in the roots. GFP fluorescence was observed in the samples infiltrated with the peptide-pDNA complex (Fig. 4D, +Bp100(KH)_9_), whereas no significant GFP signal was detected in the roots infiltrated with pDNA only (Fig. 4D, −Bp100(KH)_9_). Furthermore, the plants grown under phosphate deficiency had similar Nluc activities in both the roots and the shoots (Fig. 4E). Considering the results of the Nluc assay using the *A. thaliana* seedlings grown in the normal phosphate conditions (Fig. 1B), the length and density of the root hairs significantly affected the uptake of the peptide-pDNA complexes in the root.

## Discussion

The peptide-mediated gene delivery method has advantages other the current technology. The first advantage is applicability to various plant species by selecting optimized CPP against target tissues^28^. This can be an effective alternative to the plant species which are not infectious against *Agrobacterium*. In our previous report, based on a library containing 55 CPPs, characterization of penetrating ability for six plant species was performed^28^. As a result, there was no CPP with high permeability across all plant species and cell types, and it was revealed that optimization was necessary depending on plant type and target tissue. If CPP is optimized, all subsequent operations are common, and there is no need to change the experimental setup. The second advantage is the ability to deliver genes of interest to target organelle by using an appropriate peptide. The production of useful substances by plants has been drawing attention for a long time, but the fact that chloroplast and mitochondrial genome modification technologies have not been established has become a major bottleneck for efficient substance production^29,30^. By using the peptide method with organellar transit signals, stable and highly selective organelle transformation can be expected by the designed carrier peptide^25^. Thirdly, the peptide method, unlikely particle bombardment method or electroporation method, does not require any special equipment. From these points of view, we consider that the peptide method is a valuable technology to improve as a novel plant modification technology. In this study, we revealed the infiltration pathways of a peptide-pDNA complex, a type of non-viral gene carrier, in *N. benthamiana* and *A. thaliana*. Expression analysis of GFP revealed that the hydathodes are preferentially utilized as a complex invasion pathway in leaves (Fig. 3B,C). In the experiment using *A. thaliana*, the Nluc activity in the root was not detectable in the normal medium (Fig. 1B), whereas in the sample grown under the phosphate deficient medium, the Nluc activity was detected and statistical significance disappeared (Fig. 4E). These results indicate clearly that root hairs and hydathodes greatly contributed to the incorporation of a peptide-pDNA complex. Based on the cellular uptake behaviors of nanomaterials, root hairs, which are surface elongations of epidermal cells, contain thinner and more permeable cell walls than other root cells^31^. In addition, hydathode is structurally weaker than other tissues on the basis of the cellular uptake studies^31^. The interior of the hydathode is composed of a small group of cells with thin cell walls, and is directly connected to the vasculature of the plant^32^. These structurally weak tissues are commonly exploited as bacterial infection pathways into plants^33^.

Unlike many fungal plant pathogens that have the ability to penetrate directly from the epidermis, bacteria need to enter the leaf tissue through natural surface openings, namely the stomata, hydathodes, nectarthodes, and lenticels^34–36^. For instance, *Xanthomonas campestris* pv *campestris* (*Xcc*) is a bacterium that causes black rot disease in the Brassicaceae, and preferentially infects its hosts via their hydathodes^37,38^. Interestingly, *Xcc* is unable to infect plants via their stomatal pores even after a high-density dip inoculation of whole leaf surface^39^. Several reasons have been proposed for this, including the suggestion that the bacterium might be able to adhere more easily to the hydathode than the stomata because of the lower levels of cuticular wax associated with the hydathode pores. In the current study, our CLSM analysis showed that Cy3-labeled pDNA was present in the stomata of both *A. thaliana* and *N. benthamiana* leaves, but that GFP fluorescence around the stomata was not detected (Fig. 3A, Supplementary Fig. 1A). These results indicate that the thick cuticular wax surrounding the stomatal pores make it difficult for the peptide-pDNA complex to pass through the cell wall of mesophyll cells to reach the cytoplasm by vacuum/compression infiltration (Supplementary Fig. 2). On the other hand, our previous study showed that, when the complex solution is infiltrated into the leaf tissue using a syringe, the stomatal pores do allow the penetration of the complex^40^. Here, by immersing the intact plant seedlings in the complex solution, we revealed that the structurally weak tissues are the preferential permeation pathway of the complex.

The structural weaknesses of animals are also common infection pathways used by bacteria; for example, pathogens usually enter the body through externally accessible structures, such as the eyes, mouth, nose, urogenital openings, skin wounds, and bites^41^. The method of utilizing structural weaknesses to invade the body can be used to infiltrate both plants and animals, suggesting that fusion peptide gene carriers could be used to transform a wide range of species.

In addition, peptide-based gene carriers can permeate the plasma membrane and induce physiological activities *in vivo*. Several peptides have already been reported to function as immune responses and signaling for plant growth^42^; for example, a plant-derived immune response can be induced via a bacterial-derived elicitor. The flg22 flagellin peptide promotes stomatal pore closure to prevent the further invasion of pathogenic bacteria^43^, while antimicrobial peptides, which are biological defense substances, are known to function in the host resistance to bacteria and induce the immune response^44–46^. These facts suggest that the complex forming peptides themselves can induce physiological activity. Accordingly, future peptide complexes could be designed based on the likelihood that peptide infiltration may be controlled by the biological response.

The conventional transformation method using bacteria has several problems that the applicable plant species are limited and are restricted to modification of the nuclear genome. As an alternative to overcome these problems, the peptide-mediated delivery method is very effective due to its applicability. However, at the present time the efficiency of gene delivery to cells by peptides is far below that of bacteria. In order to enhance the delivery efficiency, it is necessary to clarify the pathway of entering the peptide complex in the plant tissue. In this study, we found that a non-viral gene delivery system using a fusion peptide, with the help of vacuum/compression infiltration, penetrates the structurally weak tissues of the plant in a similar manner to the invasion pathway of some bacteria. The structural fragility of the plant tissues is therefore crucial for the function of this gene delivery system. To improve the transformation efficiency of this system, target tissues with structural fragility must be selected. In particular, when trying to transform to new plant species, the structural characteristics of the plant tissue can be one criterion in determining the target tissue. For example, since the development of root hair is effective for incorporation of a complex, even in the case of plants which have been considered to be difficult to transform so far, it can be expected that the introduction efficiency can be enhanced by promoting root hair development. Furthermore, the similarity between the infiltration of non-viral gene carriers and bacterial pathogens suggests the possibility of improving gene delivery efficiency through peptide modification. Although the uptake pathway has been clarified by the morphological approach in this study, the molecular mechanism of complex uptake in this system remains unknown. If the molecular mechanism behind the complex uptake is clarified, we can design more effective and efficient peptides for plant transformation. Thus, the non-viral gene delivery system has more strategic flexibility than conventional gene delivery systems and may be applied to a wide variety of species.

## Materials and Methods

### Plant Materials and Growth Conditions

*Arabidopsis thaliana* (Col-0) and *Nicotiana benthamiana* seeds were sterilized in 70% ethanol for 1 min then in 10% NaClO for 15 min, after which they were rinsed three times with sterilized water to remove the NaClO. The seeds were grown on 1/2 Murashige and Skoog medium (MS Basal Medium, M5519; Sigma-Aldrich, St. Louis, MO) containing 2.5 mM MES, 1% sucrose, and 3% (w/v) agar to prevent the roots from entering the medium. After being sown on the medium, the *A. thaliana* seeds were stratified for two days in the dark at 4 °C, then grown under constant white light (100 μmol m^−2^ s^−1^) at 22 °C. The *N. benthamiana* seeds were grown in an incubator with constant white light (100 μmol m^−2^ s^−1^) and temperature at 30 °C.

### Preparation of the Peptide-pDNA Complex

Bp100(KH)_9_ (KKLFKKILKYLKHKHKHKHKHKHKHKHKH; theoretical pI/Mw: 10.81/3809.71 Da) was synthesized using the standard 9-fluorenylmethoxycarbonyl (Fmoc) solid phase peptide synthesis. The peptide was purified using high-performance liquid chromatography, and its molecular weight was confirmed using matrix-assisted laser desorption/ionization-time-of-flight mass spectrometry. Two types of plasmids, 35S-Nluc-TNOS and 35S-GFP(S65T)-TNOS^47^, respectively encoding NanoLuc® (Nluc) and GFP, were used as reporter genes. 35S-Nluc-TNOS plasmid (4102 bp) containing the P35S promoter, Nluc gene and nopaline synthase terminator (TNOS) sequence, was constructed in pGWT35S backbone^48^. Plasmids were amplified in competent DH5α *Escherichia coli* and purified using an Endofree Plasmid Giga Kit (Qiagen, Hilden, Germany). To prepare the peptide-pDNA complexes, 1.0 mg/mL Bp100(KH)_9_ peptide and 1.0 mg/mL pDNA were mixed with an N/P ratio of 0.5 or 1.0, and sterilized water was added to obtain a final volume of 500 μL. The final concentrations are 25 μg/mL for pDNA and 7.9 μg/mL (N/P = 0.5) or 15.9 μg/mL (N/P = 1.0) for peptides, respectively. The N/P ratio refers to the number of amine groups from the peptide relative to the number of phosphate groups in the pDNA. The solution was thoroughly mixed and allowed to stabilize for 30 min at 25 °C. The complexes were characterized immediately after this stabilization using a zeta potentiometer (Zetasizer Nono-ZS; Malvern Instruments, Ltd., Malvern, UK) (Supplementary Table 1). The N/P ratios with the highest transfection efficiencies were identified in the previous study^16^.

### Infiltration of the Peptide-pDNA Complex into Plant Seedlings

Ten seedlings at 7 or 10-DAG (Figs 1, 2, 3A–C and 4D,E), or five seedlings at 14-DAG (Fig. 3D–F) were immersed in a 500 μL peptide-pDNA complex solution in a 2 mL microtube, degassed at −0.1 MPa for 1 min, then pressurized at 0.1 MPa for 1 min. After this treatment, the solution was removed, and the seedlings were incubated on 1/2 MS culture plates for 24 h.

### NanoLuc Assay

The quantitative analysis of the Nluc activity was performed using a Nano-Glo® Luciferase Assay System (Promega, Madison, WI) and detected by a luminometer (GloMax® 20/20 Luminometer; Promega), according to the manufacturer’s protocol. The five or ten seedlings treated at the same time were divided into shoots and roots, each as one sample, and collected in a 1.5 mL microtubes. Then each sample was immersed in 300 μL of lysis buffer attached to the kit (Promega), and homogenized using a plastic homogenizer (#2-822-01; AS ONE, Osaka, Japan) on ice. After centrifugation at 12,000 g for 5 minutes to eliminate debris, 50 μL of supernatant was used for the assay. “Ten seedlings were treated as one sample in this assay”.

### Measurement and Analysis of Relative Stomatal Aperture

Epidermal peels were prepared as described previously^49^, with small modifications. The epidermal leaf surface was fixed to a strip of autoclave tape (#26800400; Propper, Long Island City, NY) with the abaxial side facing upwards. The stomatal aperture was evaluated by measuring the width and length of the stomatal pore observed under a microscope using ImageJ (https://imagej.nih.gov/ij/), then the relative stomatal opening was calculated as the width/length ratio.

### Confocal Laser Scanning Microscopy

The pDNA infiltration and GFP fluorescence in the leaves were observed using CLSM (LSM800; Carl Zeiss, Oberkochen, Germany). The pDNA encoding Nluc was labeled with Cy3 using a Label IT® Nucleic Acid Labeling Kit (Mirus, Madison, WI), according to the manufacturer’s protocol. Complexes of the labeled pDNA (1.0 μg/μL) and Bp100(KH)_9_ (N/P 0.5) were infiltrated into five seedlings of *A. thaliana* and *N. benthamiana*. To obtain the enlarged images in Fig. 2A, we focused on each signal, performed Z stack analysis, and displayed one of multiple images obtained in the Z direction. The fluorescence of Cy3 was observed immediately after the introduction of the complex. After the treatment, the plasma membrane was stained by immersing the seedlings in a solution of 10 μM FM4-64 (Thermo Fisher Scientific, Waltham, MA) for 15 min, after which they were washed twice with sterile water. GFP fluorescence was observed 16 h after the treatment using CLSM (LSM800; Carl Zeiss). The GFP and FM4-64 were excited at 488 nm, and the emissions were collected at 492 to 551 nm and 645 to 833 nm, respectively. Experiments were independently repeated three times. Similar results were obtained in all cases.

### Phosphate-Deficient Condition

An agar medium containing MS salts (MS Basal Salt Solution, M0529; Sigma-Aldrich) was supplemented with 2.5 mM MES, 3 mM CaCl_2_, 1.5 mM MgSO_4_, 1 mM NH_4_NO_3_, and 19 mM KNO_3_, as well as either 1 mM or 3 μM KH_2_PO_4_. When used for the Nluc assay, *A. thaliana* seeds were grown on the phosphate-deficient (3 μM KH_2_PO_4_) medium for seven days, then transferred to the normal medium (1 mM KH_2_PO_4_) for three days to recover.

### Statistical analysis

All statistical calculations were performed using XLSTAT (Addinsoft, Paris, France). Statistical differences in the Nluc assays were evaluated using a Mann-Whitney *U* test. The stomatal apertures, root hair lengths, and root hair densities were assessed using unpaired *t*-tests. The level of significance was set at *p* < 0.05 (two-tailed).

## Electronic supplementary material

Supplementary info

## Data Availability

All data generated or analyzed during this study are included in this published article (and the Supplementary Information File).

## References

[1] Belhaj K, Chaparro-Garcia A, Kamoun S, Patron NJ, Nekrasov V (2015). Editing plant genomes with CRISPR/Cas9. Curr. Opin. Biotechnol..

[2] Malzahn A, Lowder L, Qi Y (2017). Plant genome editing with TALEN and CRISPR. Cell and Bioscience.

[3] Yin K, Gao C, Qiu J-L (2017). Progress and prospects in plant genome editing. Nat. Plants.

[4] Collinge DB (2009). Cell wall appositions: the first line of defence. J. Exp. Bot..

[5] Pack DW, Hoffman AS, Pun S, Stayton PS (2005). Design and development of polymers for gene delivery. Nat. Rev. Drug Discov..

[6] Shim G, Kim M-G, Park JY, Oh Y-K (2013). Application of cationic liposomes for delivery of nucleic acids. Asian J. Pharm. Sci..

[7] Jin L, Zeng X, Liu M, Deng Y, He N (2014). Current progress in gene delivery technology based on chemical methods and nano-carriers. Theranostics.

[8] Zuris JA (2015). Cationic lipid-mediated delivery of proteins enables efficient protein-based genome editing *in vitro* and *in vivo*. Nat. Biotechnol..

[9] Liu J (2016). Cell-targeting cationic gene delivery system based on a modular design rationale. ACS Appl. Mater. Interfaces.

[10] Zhao J (2017). Multi-targeting peptides for gene carriers with high transfection efficiency. J. Mater. Chem. B.

[11] Unnamalai N, Kang BG, Lee WS (2004). Cationic oligopeptide-mediated delivery of dsRNA for post-transcriptional gene silencing in plant cells. FEBS Lett..

[12] Chugh A, Amundsen E, Eudes F (2009). Translocation of cell-penetrating peptides and delivery of their cargoes in triticale microspores. Plant Cell Rep..

[13] Zonin E (2011). TAT-Mediated Aequorin Transduction: An Alternative Approach for Effective Calcium Measurements in Plant Cells. Plant Cell Physiol..

[14] Bilichak A, Luu J, Eudes F (2015). Intracellular delivery of fluorescent protein into viable wheat microspores using cationic peptides. Front. Plant Sci..

[15] Golestanipour, A., Nikkhah, M., Aalami, A. & Hosseinkhani, S. Gene Delivery to Tobacco Root Cells with Single-Walled Carbon Nanotubes and Cell-Penetrating Fusogenic Peptides. *Mol. Biotechnol*., 10.1007/s12033-018-0120-5 (2018). 10.1007/s12033-018-0120-5 30203379

[16] Lakshmanan M, Kodama Y, Yoshizumi T, Sudesh K, Numata K (2013). Rapid and efficient gene delivery into plant cells using designed peptide carriers. Biomacromolecules.

[17] Ziemienowicz, A., Pepper, J. & Eudes, F. In417–434, 10.1007/978-1-4939-2806-4_28 (Humana Press, New York, NY, 2015).

[18] Badosa E (2007). A library of linear undecapeptides with bactericidal activity against phytopathogenic bacteria. Peptides.

[19] Chen Q-R, Zhang L, Stass SA, Mixson AJ (2000). Co-polymer of histidine and lysine markedly enhances transfection efficiency of liposomes. Gene Ther..

[20] Chuah JA, Matsugami A, Hayashi F, Numata K (2016). Self-assembled peptide-based system for mitochondrial-targeted gene delivery: functional and structural insights. Biomacromolecules.

[21] Boussif O (1995). A versatile vector for gene and oligonucleotide transfer into cells in culture and *in vivo:* polyethylenimine. Proc. Natl. Acad. Sci..

[22] Numata K, Ohtani M, Yoshizumi T, Demura T, Kodama Y (2014). Local gene silencing in plants via synthetic dsRNA and carrier peptide. Plant Biotechnol. J..

[23] Ng KK (2016). Intracellular delivery of proteins via fusion peptides in intact plants. PLoS One.

[24] Chuah J-A, Yoshizumi T, Kodama Y, Numata K (2015). Gene introduction into the mitochondria of *Arabidopsis thaliana* via peptide-based carriers. Sci. Rep..

[25] Yoshizumi, T., Oikawa, K., Chuah, J.-A., Kodama, Y. & Numata, K. Selective gene delivery for integrating exogenous DNA into plastid and mitochondrial genomes using peptide-DNA complexes. *Biomacromolecules* 8b00323, 10.1021/acs.biomac.8b00323 (2018). 10.1021/acs.biomac.8b00323 29601191

[26] Briggs WR, Huala E (1999). Blue-light photoreceptors in higher plants. Annu. Rev. Cell Dev. Biol..

[27] Raghothama KG, Karthikeyan AS (1999). Phosphate acquisition. Plant Soil.

[28] Numata K (2018). Library screening of cell-penetrating peptide for BY-2 cells, leaves of Arabidopsis, tobacco, tomato, poplar, and rice callus. Sci. Rep..

[29] Larosa V, Remacle C (2013). Transformation of the mitochondrial genome. Int. J. Dev. Biol..

[30] Adem M, Beyene D, Feyissa T (2017). Recent achievements obtained by chloroplast transformation. Plant Methods.

[31] Schwab F (2016). Barriers, pathways and processes for uptake, translocation and accumulation of nanomaterials in plants - Critical review. Nanotoxicology.

[32] Singh S (2016). Guttation: mechanism, momentum and modulation. Bot. Rev..

[33] Agrios, G. N. *Plant pathology*. (Elsevier Academic Press, 2005).

[34] Mendgen K, Hahn M, Deising H (1996). Morphogenesis and mechanisms of penetration by plant pathogenic fungi. Annu. Rev. Phytopathol..

[35] Kubicek CP, Starr TL, Glass NL (2014). Plant cell wall–degrading enzymes and their secretion in plant-pathogenic fungi. Annu. Rev. Phytopathol..

[36] Melotto M, Underwood W, He SY (2008). Role of stomata in plant innate immunity and foliar bacterial diseases. Annu. Rev. Phytopathol..

[37] Hugouvieux V, Barber CE, Daniels MJ (1998). Entry of *Xanthomonas campestris* pv. *campestris* into hydathodes of *Arabidopsis thaliana* leaves: a system for studying early infection events in bacterial pathogenesis. Mol. Plant. Microbe. Interact..

[38] Sperry JS (1983). Observations on the structure and function of hydathodes in blechnum lehmannii. Am. Fern J..

[39] Cerutti A (2017). Immunity at cauliflower hydathodes controls systemic infection by *Xanthomonas campestris* pv. *campestris*. Plant Physiol..

[40] Chuah J-A, Numata K (2018). Stimulus-responsive peptide for effective delivery and release of DNA in plants. Biomacromolecules.

[41] Cornelissen, C. N., Fisher, B. D. & Harvey, R. A. *Lippincott’s Illustrated Reviews: Microbiology (Lippincott’s Illustrated Reviews Series)*. (Lippincott Williams & Wilkins, 2012).

[42] Simon R, Dresselhaus T (2015). Peptides take centre stage in plant signaling. Preface. J. Exp. Bot..

[43] Gómez-Gómez L, Felix G, Boller T (1999). A single locus determines sensitivity to bacterial flagellin in *Arabidopsis thaliana*. Plant J..

[44] Breen S, Solomon PS, Bedon F, Vincent D (2015). Surveying the potential of secreted antimicrobial peptides to enhance plant disease resistance. Front. Plant Sci..

[45] Cole, J. N. & Nizet, V. In *Virulence Mechanisms of Bacterial Pathogens, Fifth Editio*n(ed. Kudva, T. I.) 413–443, 10.1128/microbiolspec.VMBF-0006-2015 (ASM press, 2012).

[46] Dodds PN, Rathjen JP (2010). Plant immunity: towards an integrated view of plant–pathogen interactions. Nat. Rev. Genet..

[47] Kodama Y, Wada M (2009). Simultaneous visualization of two protein complexes in a single plant cell using multicolor fluorescence complementation analysis. Plant Mol. Biol..

[48] Nakagawa T (2007). Development of series of gateway binary vectors, pGWBs, for realizing efficient construction of fusion genes for plant transformation. J. Biosci. Bioeng..

[49] Shang Y, Dai C, Lee MM, Kwak JM, Nam KH (2016). BRI1-associated receptor kinase 1 regulates guard cell ABA signaling mediated by open stomata 1 in *Arabidopsi*s. Mol. Plant.

