# Supplementary material for: Vacuum/Compression Infiltration-mediated Permeation Pathway of a Peptide-pDNA Complex as a Non-Viral Carrier for Gene Delivery in Planta
Source: Sci Rep. 2019 Jan 22;9:271. doi: 10.1038/s41598-018-36466-1 (PMC6342927; doi:10.1038/s41598-018-36466-1)
Supplement: Supplementary file 1 — Supplementary info [file 41598_2018_36466_MOESM1_ESM.pdf]

**Supplementary Information**

# Vacuum/Compression Infiltration-mediated Permeation Pathway of a Peptide-pDNA Complex as a Non-Viral Carrier for Gene Delivery *in Planta*

Keiko Midorikawa[1], Yutaka Kodama[1, 2*], Keiji Numata[1*]

**Affiliations:**

[1]Biomacromoleules Research Team, RIKEN Center for Sustainable Resource Science, 2-1 Hirosawa, Wako-shi, Saitama 351-0198, Japan.

[2]Center for Bioscience Research and Education, Utsunomiya University, Tochigi 321-8505, Japan.

*Correspondence to: keiji.numata@riken.jp, kodama@cc.utsunomiya-u.ac.jp

**Keywords:** *Arabidopsis thaliana*, cell-penetrating peptide, *Nicotiana benthamiana*, non-viral vector, transformation

**Supplementary Table 1.** Characterization and Evaluation of the Peptide-based Formulations.

| Formulation | N/P ratio | Hydrodiamic Diameter (nm) | Polydispersity Index | Zeta potential (mV) |
|---|---|---|---|---|
| BP100(KH)$_9$/35S-NLuc-TNOS | 0.5 | 226 ± 24 | 0.31 ± 0.02 | -34.8 ± 2.3 |
| BP100(KH)$_9$/35S-GFP(S65T)-TNOS | 1.0 | 150 ± 2 | 0.12 ± 0.01 | -22.8 ± 0.7 |

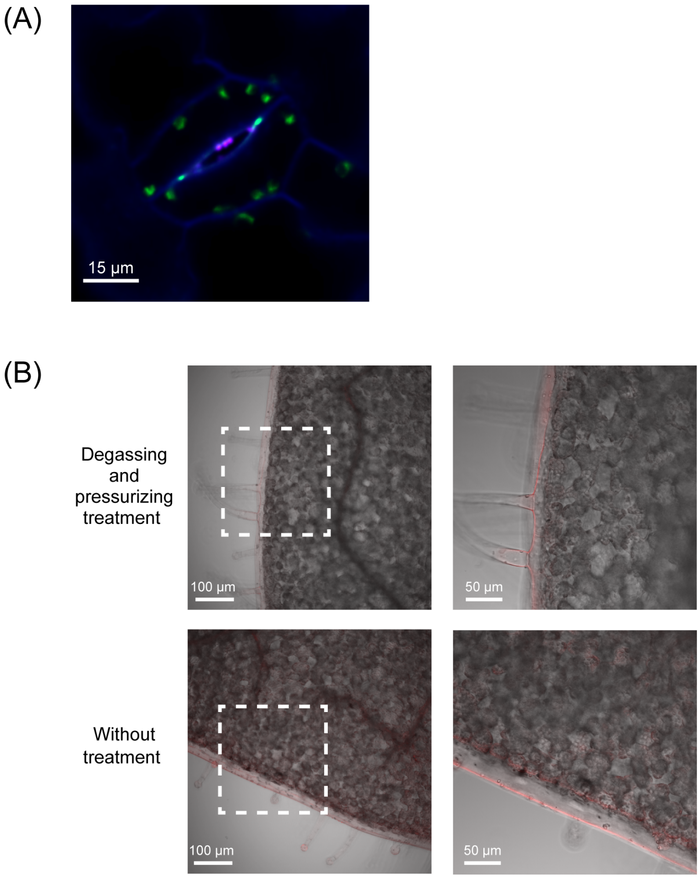


**Supplementary Figure 1.** The complex penetration to *N. benthamiana* leaf. (A) Uptake of the peptide-pDNA complex through the stomatal pore. Magenta color represents pDNA labeled with Cy3. Green is chlorophyll autofluorescence. Blue is plasma membrane stained with FM4-64. The several Cy3 signals were observed in the intercellular space. (B) Penetration of Rhodamine B staining solution in *N. benthamiana* leaves. 7-DAG seedlings of *N. benthamiana* was immersed in Rhodamine B (Wako Pure Chemical Industries, Osaka, Japan) stain (1 μg / ml), degassed for 1 min at -0.1 MPa, and pressurized at 0.1 MPa for 1 min. After treatment, the solution was removed and washed with water. The cells were observed with CLSM (LSM770, Carl Zeiss). The staining solution penetrates only to the cortical layer, even though it is infiltrated by degassing and pressurizing treatment.

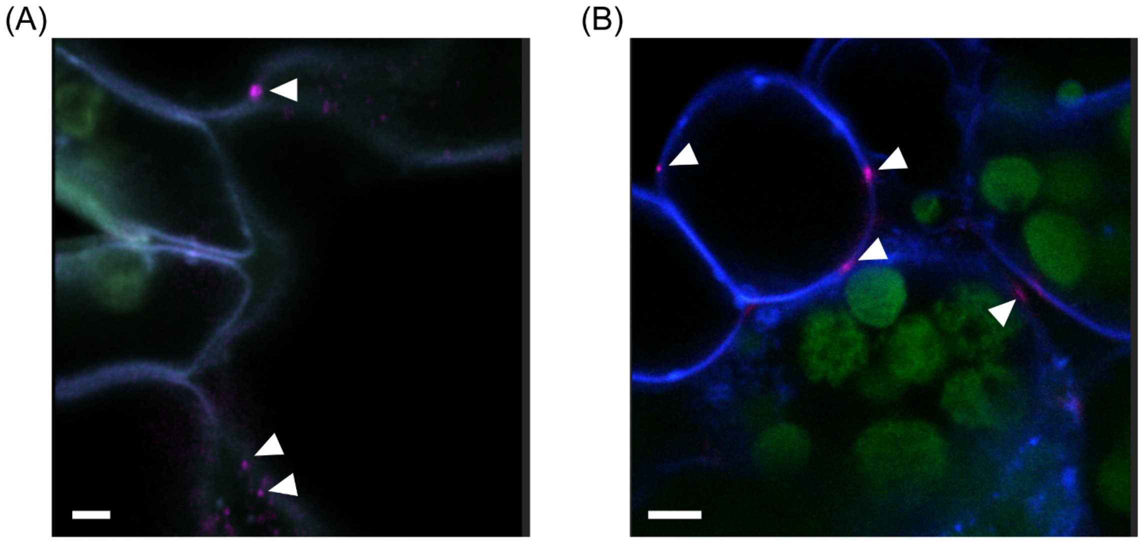

**Supplementary Figure 2.** The complex penetration to *A. thaliana* leaf tissues. The complex composed of pDNA (35S-Nluc-TNOS) labeled with Cy3 and the fusion peptide Bp100(KH)$_9$ was infiltrated by degassing and pressurizing treatment. Observation with CSLM was performed within one hour after the treatment. Magenta color represents pDNA labeled with Cy3 (white arrowhead). Green is chlorophyll autofluorescence, blue is plasma membrane stained with FM4-64. The several Cy3 signals were observed in the intercellular space. (A) Epidermal cells. (B) Mesophyll cells (bars are 5 μm).

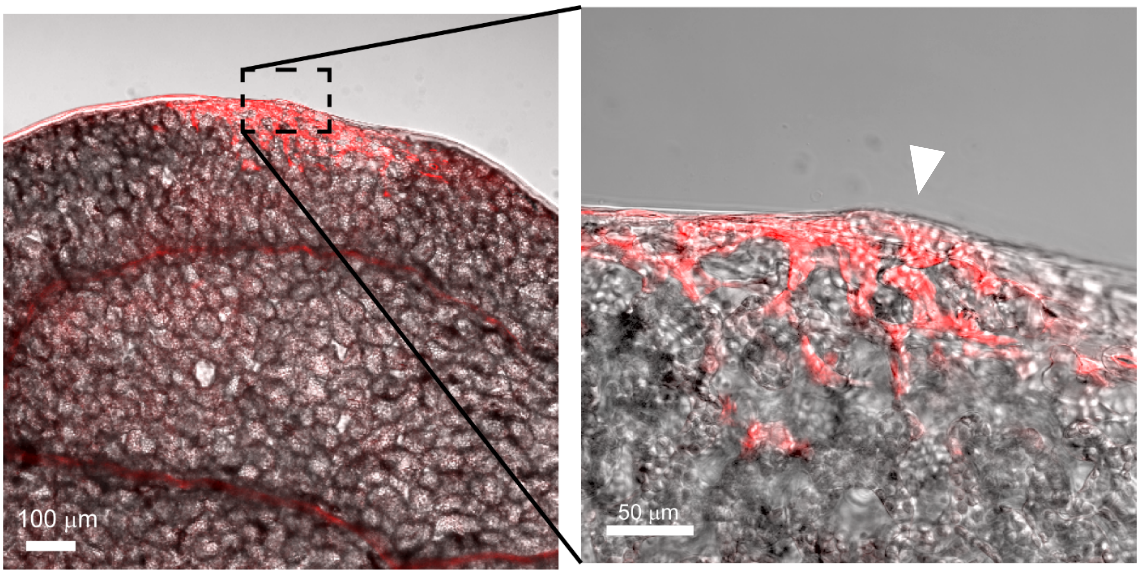


**Supplementary Figure 3.** The penetration of Rhodamine B staining solution in *A. thaliana* leaf (7-DAG). The staining solution infiltrated into the leaves from hydathode. Rhodamine staining was performed in the same way as Supplementary Figure 1B.

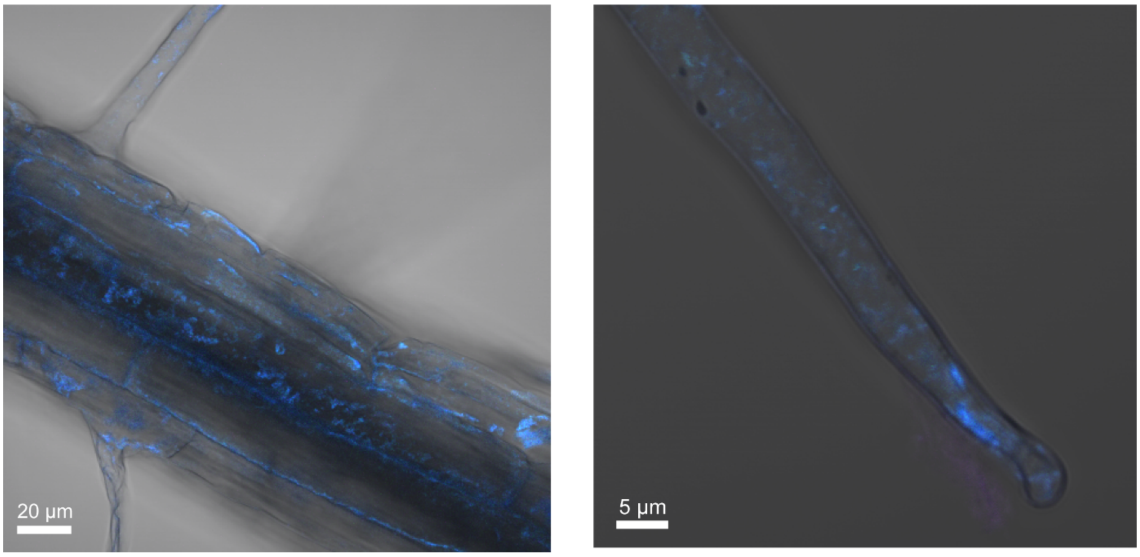


**Supplementary Figure 4.** The complex penetration in *A. thaliana* primary root (left) and root hair (right). 7-DAG seedlings were infiltrated with the complex consisting of Cy3-labeled pDNA (35S-Nluc-TNOS) and the fusion peptide, Bp100(KH)$_9$. Blue fluorescence indicates plasma membrane or endosome stained with FM4-64. Observation with CSLM was performed within one hour after the degassing and pressurizing treatment, but no Cy3 signal (magenta) was detected around the root.

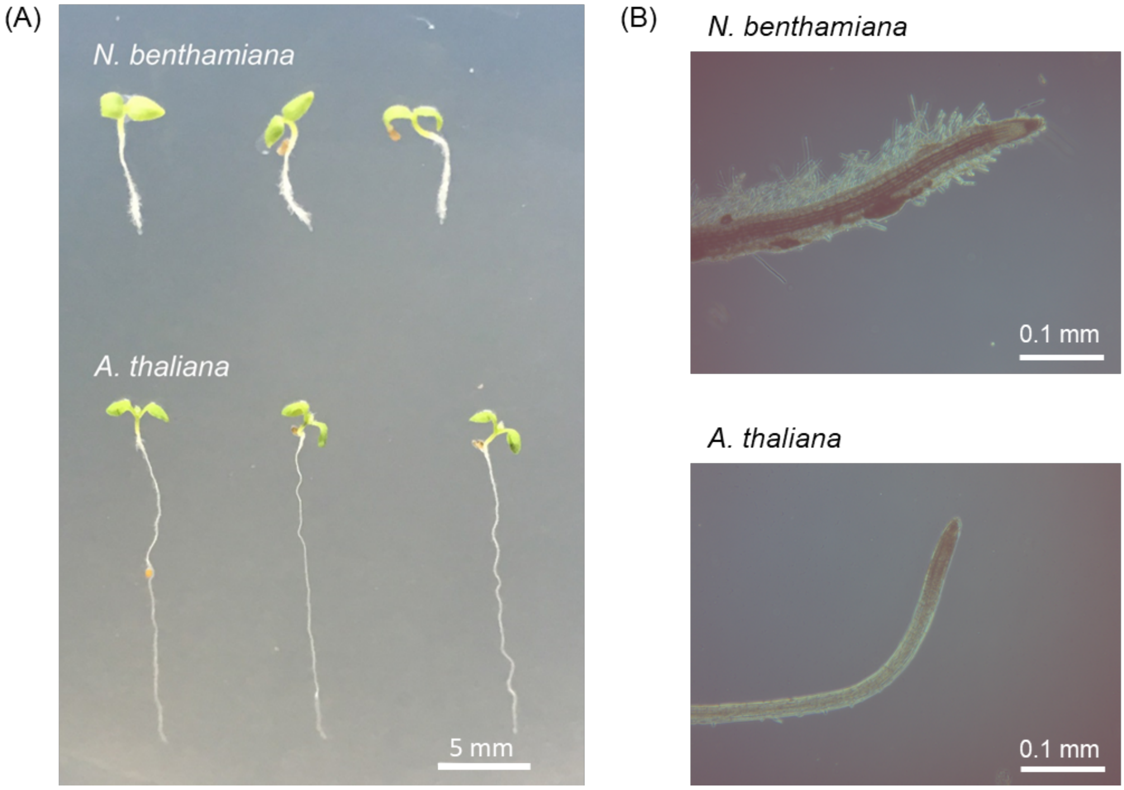


**Supplementary Figure 5.** Root morphology of *A. thaliana* and *N. benthamiana*. 7-DAG seedlings (A) and root tips (B) of *N. benthamiana* and *A. thaliana*. *N. benthamiana* is more developed in root hair.
